# Remote patient monitoring in autoimmune related interstitial lung diseases: a narrative review

**DOI:** 10.3389/fimmu.2025.1643176

**Published:** 2025-12-15

**Authors:** Malik A. Althobiani, Maryam Almoagal

**Affiliations:** 1 Department of Respiratory Therapy, Faculty of Medical Rehabilitation Sciences, King Abdulaziz University, Jeddah, Saudi Arabia; 2 Respiratory Therapy Unit, King Abdulaziz University Hospital, Jeddah, Saudi Arabia; 3 Respiratory Therapy Department, College of Applied Medical Sciences, King Saud Bin Abdulaziz University for Health Sciences, Al Ahsa, Saudi Arabia; 4 Respiratory Medicine, Division of Medicine, University College London, London, United Kingdom

**Keywords:** interstitial lung disease (ILD), autoimmune rheumatic diseases, remote monitor, wearables, artificial intelligence - AI, machine learning

## Abstract

Autoimmune related interstitial lung disease can worsen between clinic visits, and episodic assessment may miss clinically important change. Digital health extends observation into daily life through home spirometry, wearable sensors, application based patient reported outcomes, and therapist supported telerehabilitation. This Review synthesizes recent evidence on feasibility and adherence, data quality and agreement with clinic assessments, patient experience and safety, and service integration for remote monitoring in autoimmune related interstitial lung disease. Device derived signals and patient generated health data show useful agreement with clinic measures when interpreted across repeated time points, and remote monitoring data can reveal actionable trends and support rehabilitation and self-management. Important limitations remain, including variability and artifacts, missing data, uneven interoperability, workload implications for services, and inequities in digital access. We outline a practical workflow for adoption that includes enrolment, training, quality checks, alert thresholds, and escalation to the multidisciplinary team, with attention to privacy, cost, and record integration. Remote monitoring can complement standard care by increasing observation frequency and patient support. Priorities for the field are to define clinically meaningful digital endpoints, evaluate effects on outcomes and use of resources, and develop strategies that sustain long term engagement.

## Introduction

Autoimmune related interstitial lung disease (AR-ILD) presents a significant management challenge due to its unpredictable disease trajectories and the limitations of traditional, episodic clinical assessments. The need for more frequent and continuous monitoring to detect early disease progression has led to a growing interest in digital health technologies. This review explores the role of remote monitoring devices, smartphone applications, and wearable sensors in transforming the care of patients with AR-ILD (1–4).

To address the limitations of this episodic care model, digital health technologies offer a novel approach to capture day-to-day disease fluctuations and empower proactive management. Therefore, this narrative review synthesizes the recent evidence on the use of digital medicine and remote monitoring in AR-ILD. Specifically, we examine: 1) feasibility and patient adherence; 2) data quality and agreement with clinic assessment; 3) patient experience and safety; and 4) integrating challenges and opportunities, including practical workflows and evaluation frameworks.

**Figure 1:**
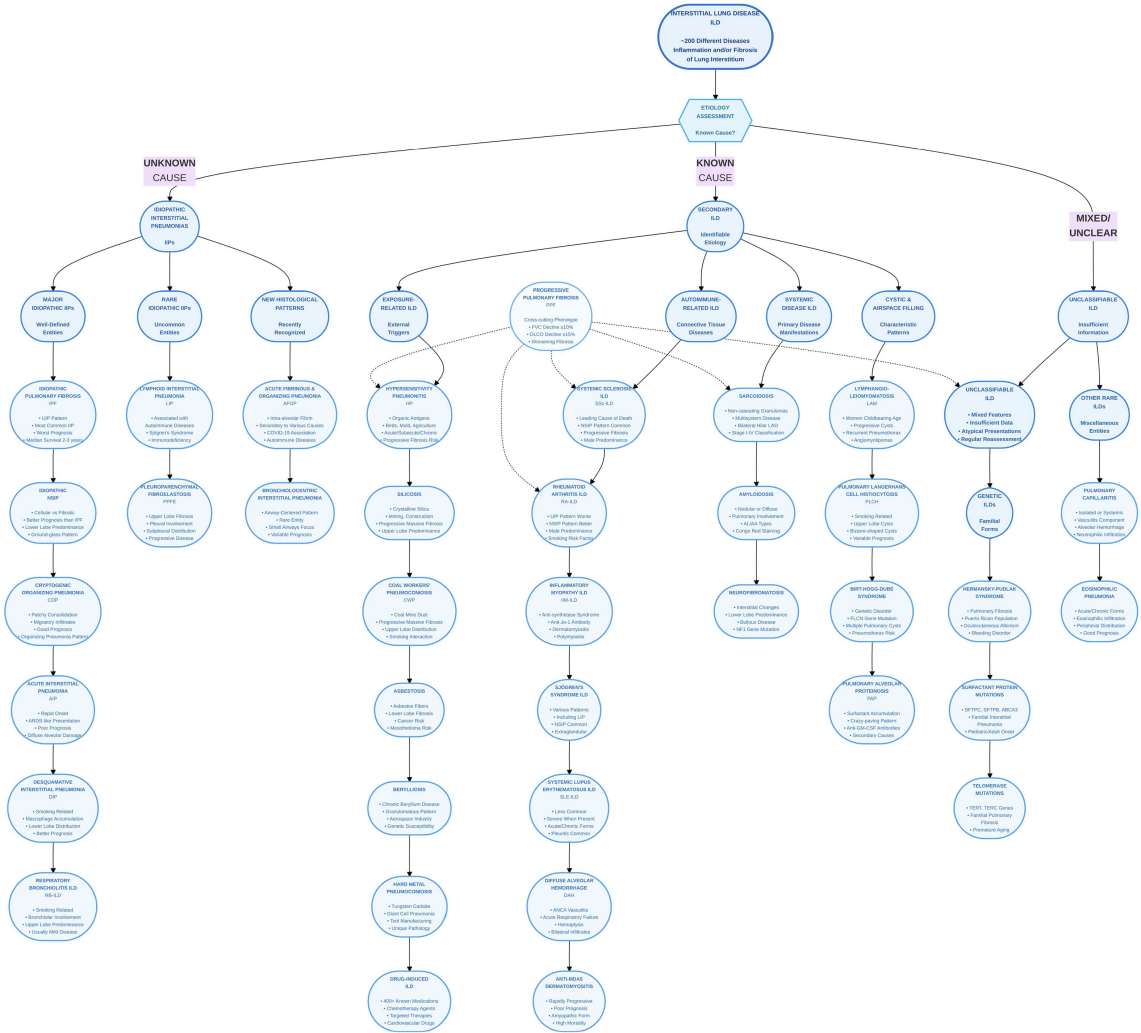
Classification of interstitial lung diseases (ILDs) by etiology.

**Figure 1**: The figure shows how ILDs can be grouped by cause: those of unknown origin (including the idiopathic interstitial pneumonias), those with identifiable triggers (including Autoimmune related ILD, the focus of this review), and cases that remain unclassifiable. The heterogeneity evident in this classification creates practical challenges for monitoring, since different ILD subtypes follow different natural histories and may require distinct surveillance approaches that episodic clinic assessments struggle to provide.

The case for continuous monitoring becomes clearer when we consider the clinical characteristics of Autoimmune related ILD itself. Rheumatoid arthritis, systemic sclerosis, the idiopathic inflammatory myopathies, Sjögren’s disease, and ANCA-associated vasculitis can all cause serious lung involvement, with ILD representing the pulmonary manifestation most strongly associated with mortality (5, 6).These conditions encompass a range of subtypes, RA-ILD, SSc-ILD, SLE-ILD, DM-ILD, ASyS-ILD, and others, each with its own epidemiology and prognosis (7). Rheumatoid arthritis-associated ILD illustrates the stakes: affected patients face a threefold increase in mortality compared with those who have RA without lung disease, and many develop progressive pulmonary fibrosis characterized by declining lung function and worsening symptoms (8, 9). A recent prospective study found that more than one-third of RA-ILD patients met criteria for progressive pulmonary fibrosis over three years, with baseline biomarkers like KL-6 and hSP-D helping to identify those at highest risk (10). Yet the central challenge is variability. Some patients remain stable for years; others deteriorate rapidly. Distinguishing between these trajectories early enough to intervene requires more frequent observation than quarterly clinic visits can provide (11).

**Figure 2:**
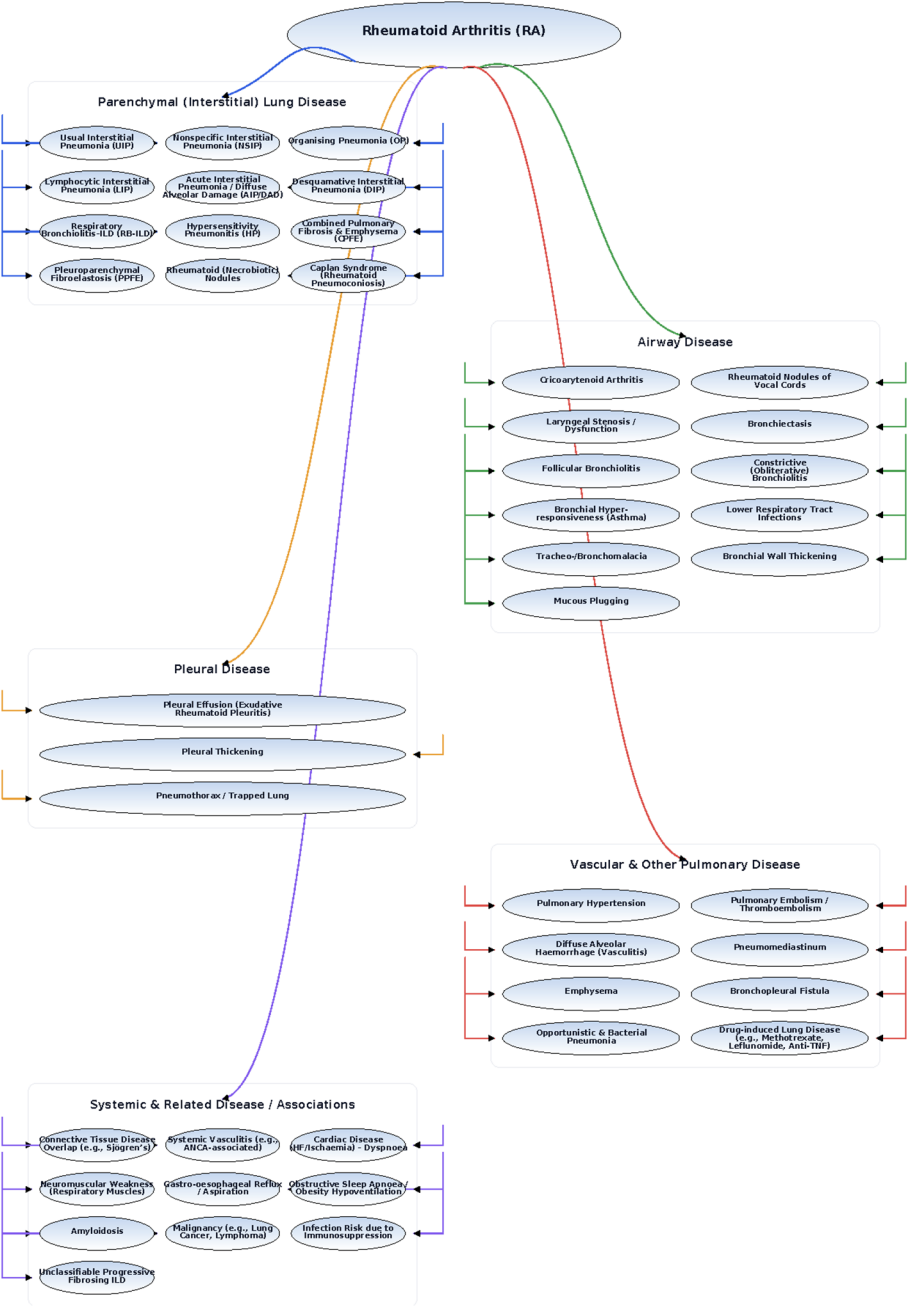
Conceptual framework and spectrum of pulmonary disease in rheumatoid arthritis–associated ILD (RA-ILD).

**Figure 2** emphasizes the distinct position of rheumatoid arthritis–associated ILD within the broader spectrum. It highlights the central role of RA-ILD in linking systemic autoimmune disease with pulmonary manifestations and demonstrates how recognition of the underlying rheumatologic context is essential for accurate diagnosis, prognostic stratification, and tailored management within the multidisciplinary evaluation of ILD. Rheumatoid arthritis affects the lungs in multiple ways, parenchymal disease (including UIP, NSIP, and organizing pneumonia patterns), airway involvement, pleural complications, and vascular abnormalities. The parenchymal ILDs carry the greatest prognostic weight, but their clinical course is highly variable and difficult to predict. This complexity, spanning multiple organ compartments and histological patterns, illustrates why infrequent assessments often miss important changes and why continuous monitoring might offer clinical value.

Current diagnostic and monitoring practices rely on well-established tools: high-resolution CT scanning for anatomical detail, pulmonary function tests to quantify physiological impairment, exercise testing to assess functional capacity, and questionnaires to capture patient-reported symptoms (12, 13). Each has limitations when used for longitudinal monitoring. HRCT remains the reference standard for identifying patterns like UIP, NSIP, and organizing pneumonia, but radiation exposure and cost make serial imaging impractical for routine surveillance (14). Spirometry, particularly FVC and DLCO measurements are central to clinical decision-making and have proven value in tracking disease progression, yet they represent single timepoints that can be affected by day-to-day variability, testing technique, and transient factors unrelated to underlying disease (15, 16). Studies of home spirometry in ILD have shown reasonable correlation with clinic measurements, though home values tend to run lower and variability can complicate interpretation (17–19). The 6-minute walk test adds functional information but can be confounded in patients with rheumatic diseases by joint symptoms or Raynaud’s phenomenon affecting oximetry readings (20). Beyond these technical considerations, clinic-based monitoring creates access barriers, particularly for patients in rural areas or those with limited mobility, and imposes time and travel burdens on people already managing complex chronic illness (21).

**Figure 3:**
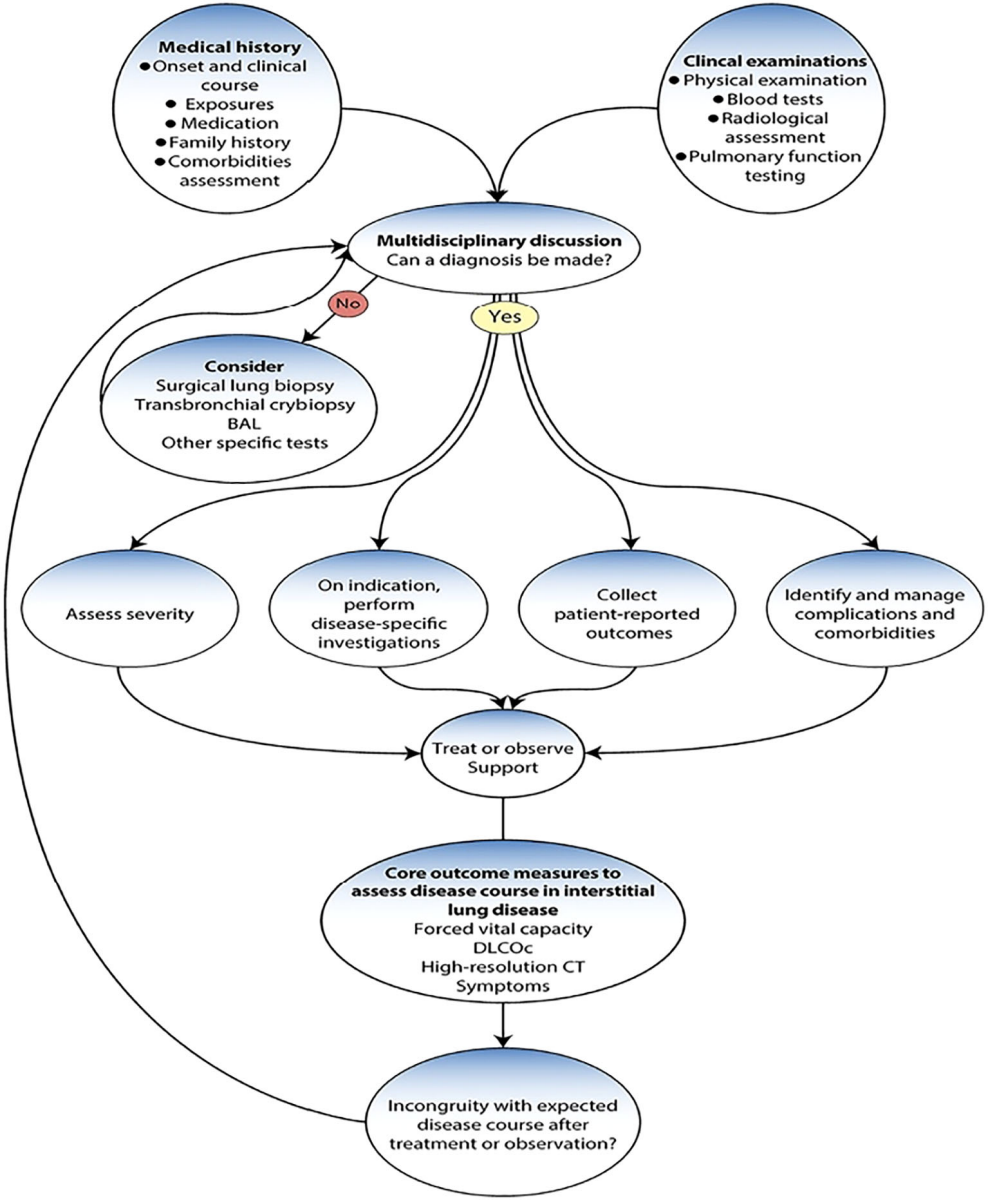
Multidisciplinary pathway for ILD diagnosis and management.

**Figure 3**: Multidisciplinary pathway for ILD diagnosis and management. The flowchart outlines how patients move through evaluation (history, examination, imaging, pulmonary function testing) to diagnosis and then into a cycle of reassessment. While this framework works well for establishing diagnosis, the typical 3–6 month interval between reassessments creates gaps where subclinical progression may go unnoticed. These gaps represent opportunities where continuous monitoring through digital tools could potentially improve detection of disease activity and inform earlier treatment adjustments.

Treatment decisions in Autoimmune related ILD are rarely straightforward. The 2023 ACR/CHEST guideline offers 35 recommendations for managing ILD in systemic autoimmune rheumatic diseases, reflecting both the evidence base and the complexity involved (5). Choices depend on the underlying rheumatic disease, whether the predominant process is inflammatory or fibrotic, and the anticipated disease course. Options include immunosuppression with agents like mycophenolate or cyclophosphamide for inflammatory phenotypes, antifibrotic drugs such as nintedanib or pirfenidone for progressive fibrosis, or combinations of these approaches, alongside supportive measures including pulmonary rehabilitation, oxygen therapy, and symptom management (5, 22, 23). Recent real-world data suggest that antifibrotics can stabilize lung function in progressive RA-ILD, though tolerability varies (24). What complicates matters is the absence of consensus on when to start or escalate therapy, leaving clinicians to make judgment calls based on incomplete information (25). Effective monitoring matters because it informs these decisions, detecting early progression, assessing treatment response, identifying acute exacerbations. The problem is that waiting three to six months between assessments may mean missing the window when intervention would be most effective (26).

**Figure 4:**
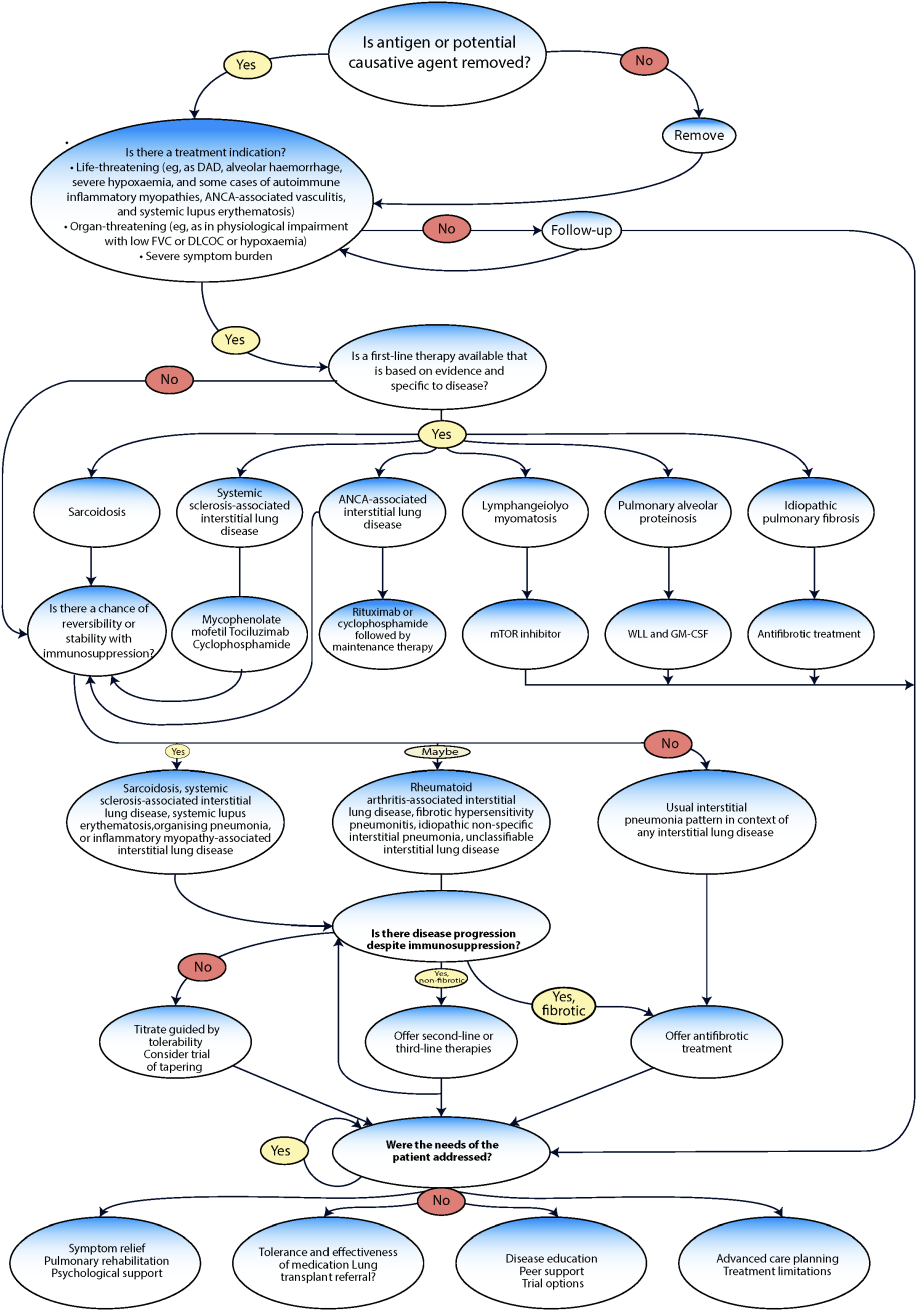
Multidisciplinary pathway for ILD diagnosis and management.

**Figure 4** shows the decision pathway from initial assessment through first-line therapy, evaluation of response, and consideration of second-line or antifibrotic treatment. Each branch point depends on timely recognition of disease behavior, whether the patient is stable, progressing despite therapy, or developing a fibrotic phenotype. Traditional monitoring intervals can delay these determinations, whereas continuous data from digital monitoring could potentially support more responsive, individualized treatment decisions and better integration of supportive care, transplant evaluation, and advance planning.

This review examines the evidence for digital medicine and remote monitoring in Autoimmune related ILD. We assess the feasibility of these technologies and whether patients actually use them consistently, evaluate how data from home devices compare with traditional clinic measurements, consider patient perspectives and safety, and discuss the practical challenges of implementation. Our aim is to provide a realistic appraisal of where remote monitoring stands today and what would be needed to make it work in routine care for patients with Autoimmune related ILD.

## Digital health and remote monitoring

Since RA-ILD poses a significant challenge to the medical field, its close monitoring plays a crucial role in improving quality of life (QoL). Essentially, monitoring signs and symptoms as indicators of progression enables the assessment of severity, evaluation of treatment responses, and early detection of complications, thereby improving management. RA-ILD monitoring is a multimodality process that encompasses all the disease aspects, including measurements of pulmonary function, exercise capacity, QoL, symptom burden, adverse events, comorbidities, radiological imaging, palliative care needs, and lung transplantation (27, 28).

Traditionally, the monitoring of RA-ILD patients has been clinic centered, requiring frequent hospital visits, undergoing numerous tests, including imaging, PFTs, and face-to-face symptom evaluation. Among the PFT standard monitoring practices, FVC is the most important clinical indicator of ILD progression. It provides a detailed measurement of lung volumes over time using a spirometry device. Additionally, the DLCO evaluates gas exchange diffusion and the integrity of the alveolar-capillary membrane. It is a high-quality indicator of disease severity and worsening fibrosis. Furthermore, oxygen saturation (SpO2) monitoring assesses the breathing quality and gas exchange integrity. Functional exercise capacity is another essential standard monitoring indicator, primarily evaluated using the 6MWT. Unfortunately, it is not beneficial for all ILDs, as it is more challenging in rheumatic disease patients because of their extra-pulmonary problems, such as joint pain, impacting physical activities and ambulation. Also, difficulties in performing hypoxic evaluation during the test in patients with Raynaud’s phenomenon may be confounded by falsely low SpO2 due to reduced digital perfusion (28). Additionally, patient-reported outcomes (PROs) are used to assess QoL via questionnaires. For instance, Saint George’s Respiratory Questionnaire (SGRQ), assessing reliability, validity, and responsiveness in CTD-ILD patients (29). Also, the King’s Brief Interstitial Lung Disease questionnaire (K-BILD) and the University of California San Diego Shortness of Breath Questionnaire (UCSD-SOBQ), which assesses functional status and dyspnea as a major determinant of QoL (27, 28).

Overall, the disease types and behaviors specify the needed monitoring devices. However, some ILD complex types require a specific monitoring plan. For example, SS-ILD patients must be monitored using multiple methods, including symptom assessment, PFTs, exercise-induced blood oxygen desaturation, and, where appropriate, HRCT (30, 31). However, there are no international guidelines regarding follow-up radiological imaging for progressive fibrosing ILDs, but it is typically performed every 12–18 months if needed (28).

The frequency of clinical visits depends on the disease behavior, patient preferences, and any assessment needed to guide the follow-up strategies. For example, outpatient regular visits are initially planned every 3–4 months, and stable patients with few symptoms have longer intervals up to 6–12 months (27, 28).

Nevertheless, clinic-based monitoring has several limitations affecting the overall management. With infrequent long-interval assessments, there is a higher probability of missing early exacerbations and disease progression outside the clinic. Alongside the travel burden in some patients from rural countries with access limitations to clinic care, such as long distances, time, and cost. Therefore, RA-ILD home monitoring is crucial to ensure continuous close patient monitoring, reduce adverse events, minimize the disease burden, educate patients, and promote self-management. Recent advancements in digital health have enabled a paradigm shift toward home-based remote monitoring, offering continuous data collection, real-time symptom tracking, monitoring of physiological parameters and functional status, with improved disease management and reduced hospital dependency.

**Figure 5:**
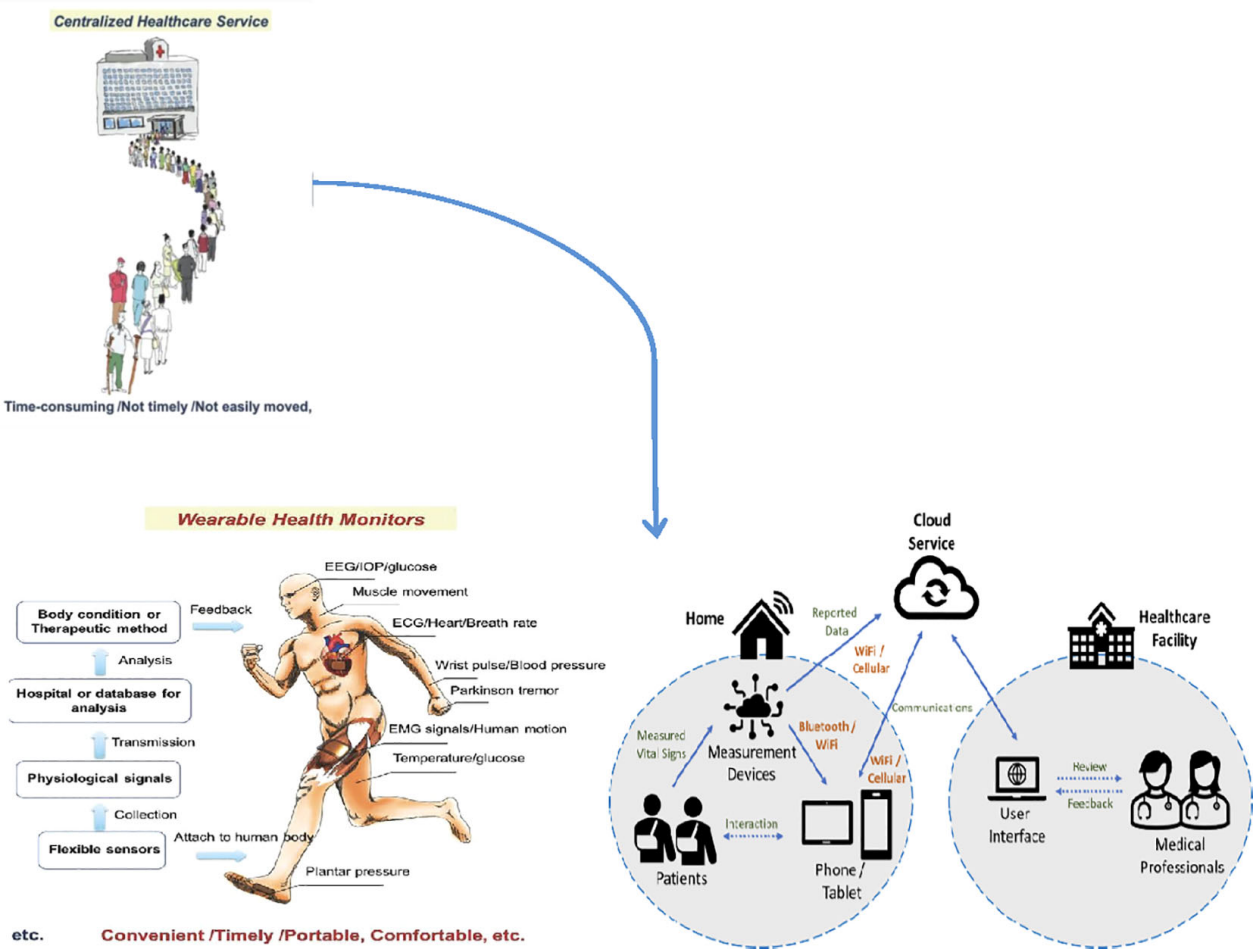
Illustration of an IoHT-based solution architecture, adapted for AR-ILD remote monitoring (e.g., biosensors for pulmonary function data flowing to gateways and cloud servers for clinician review) (32–34).

To address these limitations, digital health technologies can leverage Internet of Health Things (IoHT) architectures for seamless integration of remote monitoring tools. For instance, an IoHT-based solution architecture can facilitate data flow from patient sensors to cloud storage and healthcare providers, enabling real-time tracking of vital signs like SpO2 and FVC in AR-ILD patients (**Figure 5**). Smartphone-based solutions further enhance this by storing and processing electrophysiological data, aligning with app-based PROs and telerehabilitation for improved patient adherence and experience.

**Figure 6:**
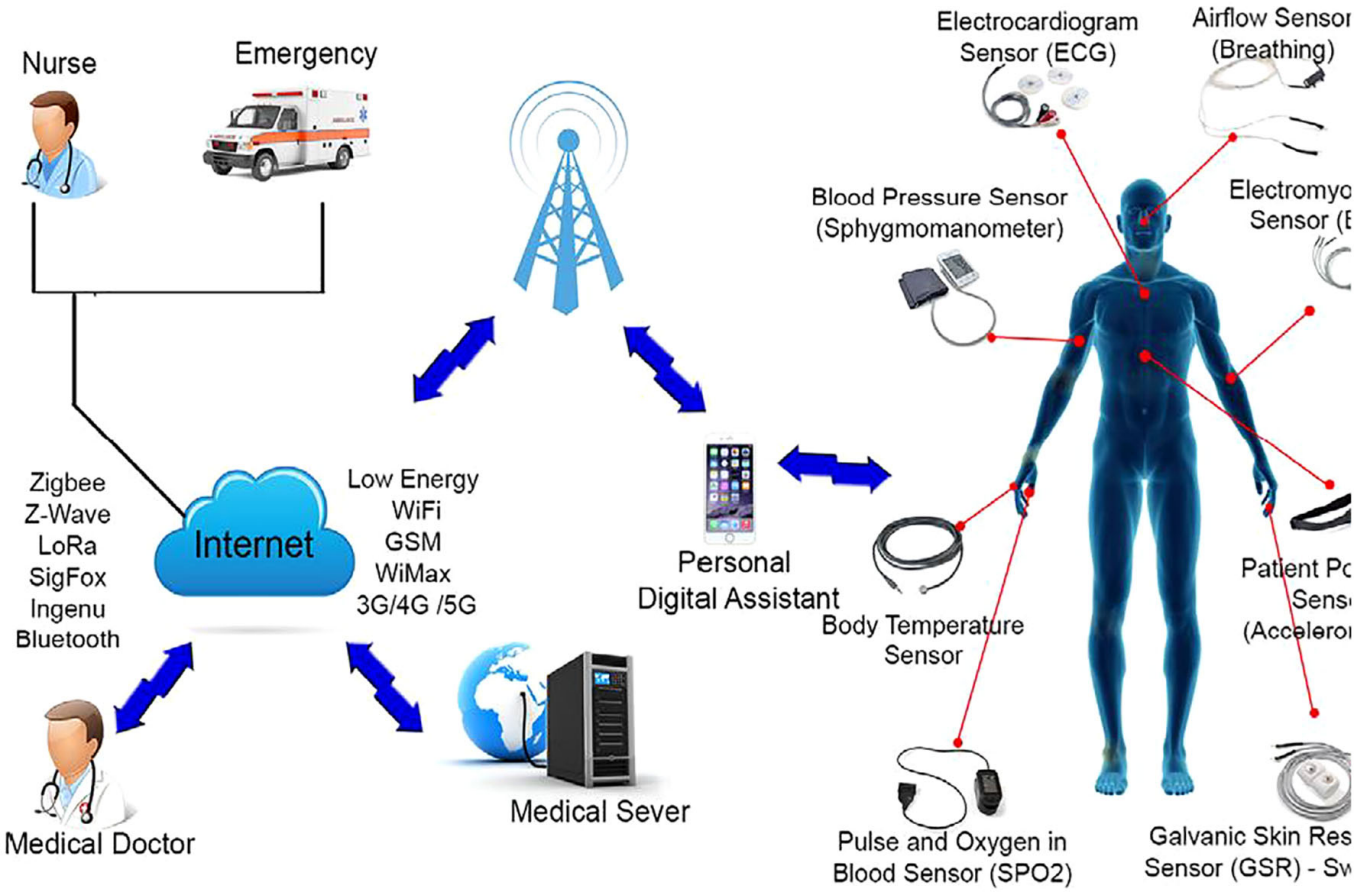
Tiered architecture for remote healthcare monitoring in IoHT, illustrating patient-assistant-medical server integration for AR-ILD vital sign tracking (32).

This architecture supports remote healthcare monitoring systems, where tiered components (patient devices, assistants, and medical servers) ensure data quality and agreement with clinic assessments, as seen in studies like REMOTE **Figure 6**.

**Figure 7:**
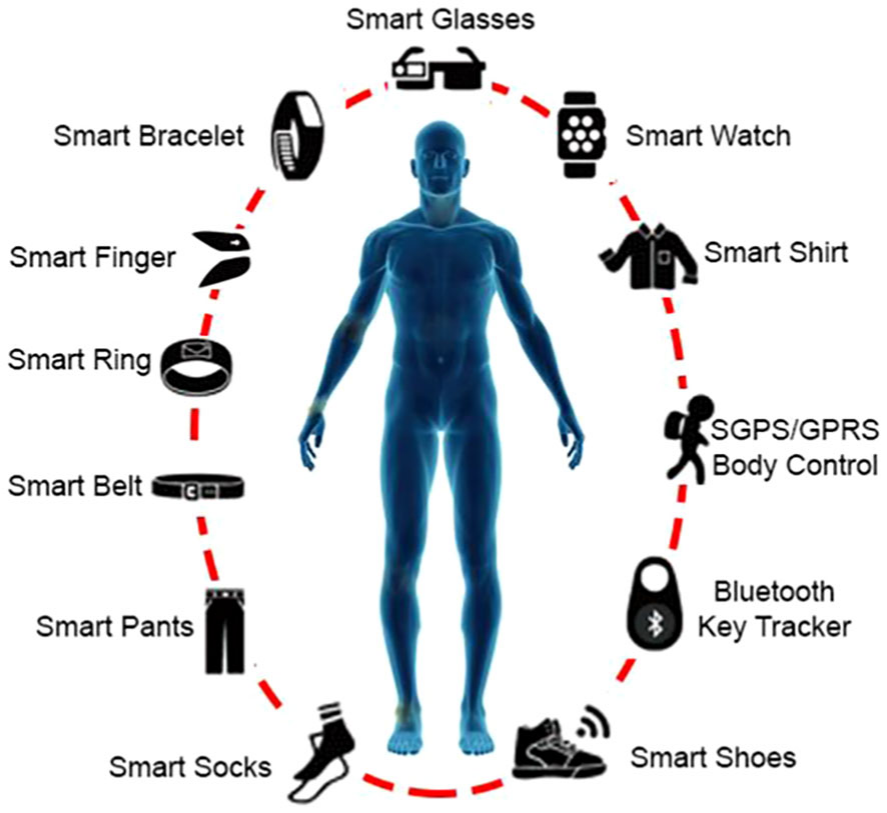
Different types of wearable technology in IoHT for monitoring vital signs like heart rate and oxygen saturation in AR-ILD.

**Figure 8:**
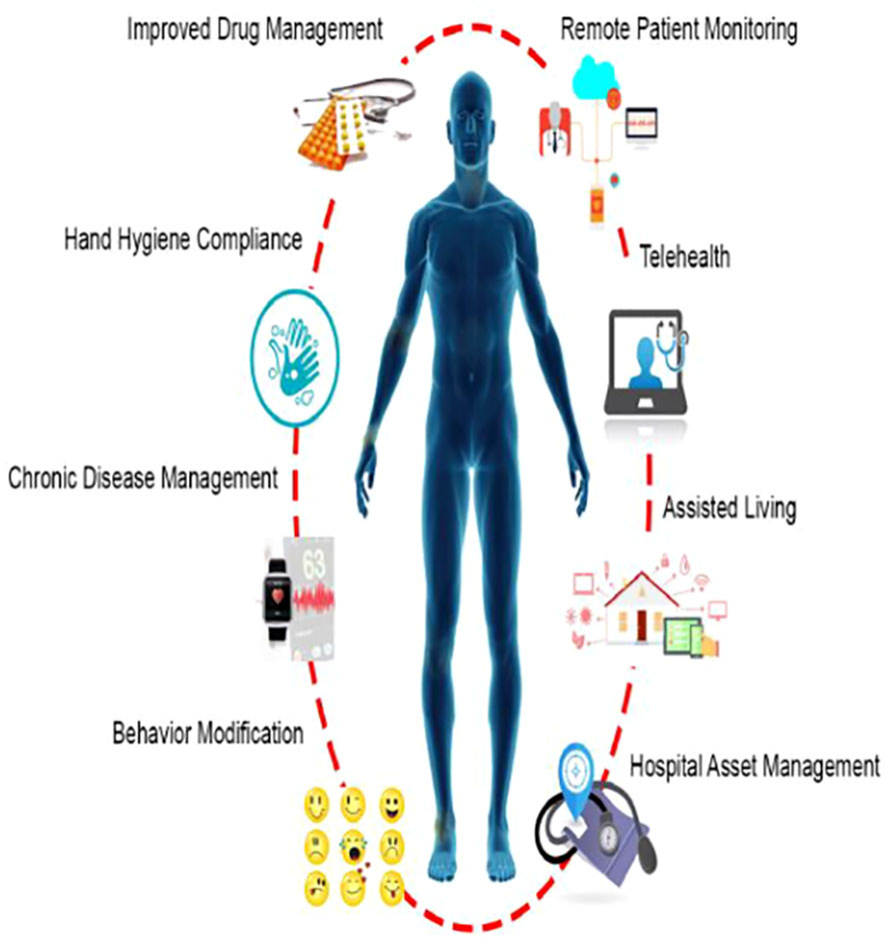
Illustration of an ambient assisted living system in IoHT, adapted for elderly AR-ILD patients to enhance safety and autonomy (32).

For patients with mobility issues, ambient assisted living (AAL) systems in IoHT can provide home-based support, such as fall detection or environmental monitoring, complementing AR-ILD self-management **Figure 7**. Finally, wearable technologies offer diverse options for vital sign monitoring, directly supporting home spirometry and SpO2 tracking in AR-ILD **Figure 8**.

Also, pulmonary rehabilitation has shown significant improvement in exercise capacity, QoL, and dyspnea, and is internationally recommended for chronic RA-ILD patients. However, RA-ILD patients, among others, have low participation proportions due to limited service availability, difficulties with traveling for some rural-resident patients, and compromised mobility (27). Therefore, telerehabilitation promises more ease in overcoming these issues. A study investigating home-based pulmonary rehabilitation conducted by Duke et al. in 2023, revealed that 50% of their cohort experienced improvement in their mean steps associated with dyspnea and respiratory-related QoL, with 33.33% reporting clinical dyspnea improvement based on the modified Medical Research Council (35).

## Clinical significance of remote monitoring on healthcare systems

The most common remote monitoring device is home-based spirometry, which is the typical standard device used to provide accurate and precise PFT parameters due to its feasibility and accuracy compared to the conventional in-clinic spirometry, as it enables daily and weekly FVC tracking for remotely monitoring ILD (36). It can be used alone, such as in real-time wireless home spirometry, or in conjunction with applications like the patientMpower home-monitoring application (37). Although some studies have shown high variability in parameters, as seen in the study by Maher et al. (36) Other studies, like Moor CC, et al., reported that “home spirometry measurements highly correlate with in-hospital measurements of FVC (r = 0.94; p < 0.001), but with slightly lower readings at home” (38). A recent study by Velauthapillai et al. reported a high sensitivity percentage (60%) of home spirometry in detecting 5% or more of FVC predicted decline, with 87% specificity observed in SSc-ILD patients, showing no differences between hospital and home assessments (39).

Additionally, wearable smart devices and smartphone applications are increasingly emerging but are not yet standard practice in the UK. These smart devices have been beneficial in tracking the patient’s physical activities and SpO2, such as Fitbit and Apple Watches. Studies evaluating feasibility and adherence to home-spirometry show a high percentage, 25%-98.8% (17, 40). For example, A study by Edwards et al. evaluating the 1-year experience of 36 pulmonary fibrotic patients using home-based spirometry and patientMpower application with self-reporting and usual care, results showed most participants (93%) stated “*they would recommend patientMpower to others and wanted to continue using it after the initial 6 weeks*”, with addressing that it helped in taking their correct medication doses, achieve their exercise goals, and positively affected their well-being and daily life (40).

A recent study by Jonassen et al. published their findings on how remote monitoring can significantly support the patients’ emotional needs, which substantially affect QoL. Their finding indicates that remote monitoring usage as a personalized approach has not been driven by socioeconomic and cognitive needs alone, but also by emotional needs, which are neglected but highly important. Additionally, it revealed positive relationships between patients and healthcare providers outside the hospital, which addressed their critical human needs to be heard, empowered them to take control over their health, and made them feel cared for. As stated by their participants, “*There is a lot more personalization, which again makes it so much more about you.*” Specifically helpful for elderly patients as it counteracts their loneliness: “*I know of a lot of elderly people who feel, especially with the advent of the pandemic, have felt very isolated, feeling like you have that kind of regular support could make all the difference in the world*” (41).

Overall, high patient satisfaction with remote monitoring is evident, as a recent systematic review studying the role of home monitoring in ILD patients included 13 studies with a total of 968 patients, showing a high adherence (75%), with a significant association between home spirometry and in-clinic spirometry (42).

To ensure high-quality remote monitoring, electronic-health technologies, such as home spirometry and oximetry, have been continuously enhanced to meet the RA-ILD monitoring requirements. For example, targeting a more straightforward setup, improved usability, and higher accuracy to guarantee patients’ convenience and adherence with the lowest technical issues.

**Table 1** provides a comprehensive overview of the currently used home monitoring devices, based on previous studies indicating that home monitoring is feasible and acceptable in RA-ILD patients, which holds promise for earlier detection of progression or acute exacerbations, enabling timely therapeutic interventions. The Air-smart handheld mobile spirometer connected to a standard smartphone shows measurements with a 97.6% sensitivity and a 74.4% specificity (43). Additionally, offering insights into patients’ experiences using such tools at home highlights how symptom tracking may influence self-awareness by providing patients with immediate feedback on respiratory patterns and disease management, which is central to the study’s exploration of patient-centered models of monitoring.

**Table 1:** Technical and clinical insights.

Device & monitoring modality	Technical features	Clinical use	Data transmission	Usability	Accuracy	Adherence	Limitations
Home spirometry • Spirobank Smart (MIR)^.42,43^ • Air-Smart Spirometer (mobile spirometer) (43).	Bluetooth, mobile app integration, FVC/FEV1 tracking	Detect FVC decline in IPF & CT-ILD	ArtiQ AI software/Smartphone application to cloud	Moderate, requires training	High	Moderate, affected by patient effort	Requires proper technique
Wearable oximeter • Nonin Medical (44). • COVID Oximetry@home (45).	o Continuous SpO_2_ o Motion detection	Nocturnal desaturation.	Bluetooth to application/cloud	High, passive wear	Moderate	High, passive data collection	Not FDA-approved
Multi-modal (spirometry + symptom tracking) • PatientMpower (40, 46).	o PROs. o Portable spirometer o Digital diary	Longitudinal monitoring, early exacerbation signs	Encrypted cloud sync	Moderate, app-device coordination required	Moderate to high	Variable, depends on patient engagement	Device calibration issues
Acoustic (cough monitoring) • ResAppDx (47). • Leicester Cough Monitor (17). • VitaloJAK (17). HealthMode Cough (48).	Smartphone mic-based AI analysis	Cough pattern tracking.	Mobile application	High, app-based, non-invasive	Early-stage – under evaluation	High, non-intrusive use	Limited specificity
Tele-rehabilitation (physical activity monitoring • ActiGraph (17). • Pensacola (17). • FL (17). • VAPA platform (49).	use signal transduction strategies to detect SpO_2_ and heart rate.	As 6MWT.	Mobile application	High, app-based, non-invasive	Moderate	Moderate to high.	Lack of accuracy in assessing muscle tone and movement.

## Usability, acceptability, and experience

Several recent studies reveal great patient usability and acceptance of their remote monitoring experience. As seen from Velauthapillai A, et al.’s study, which assesses pulmonary function home monitoring of systemic sclerosis patients and clearly stating their patients’ confidence sentences in this monitoring method, as a female participant, who aged 56-year-old with 9 months of remote monitoring experience said: “*I think weekly measurements give a better overview than a one-time measurement once every 3 months*”. Also, the patients’ high satisfaction was expressed by recommendations for home monitoring, as another female participant, aged 59-years-old who had a full year of remote monitoring experience stated: “*I would really recommend it as part of regular healthcare*” (50). Glenn et al. conducted a pilot study to assess the smartphone application usability, showing 76.0% of participants agreed or strongly agreed that the app was easy to use, and 74.8% found the method well-organized with accessible information (51).

However, remote monitoring is not suitable for all patients. As some issues may limit its use, such as patient disabilities like hearing loss or difficulties in performing the test due to excessive saliva production, or technical issues affected by network limitations, malfunctioning application, or inadequate knowledge, highlighting the importance of clear instructions (27, 50). As seen in some older participants struggled with the use of applications due to limited digital literacy, which had been anticipated also by some younger participants (50).

## Challenges in remote monitoring

**Figure 9:**
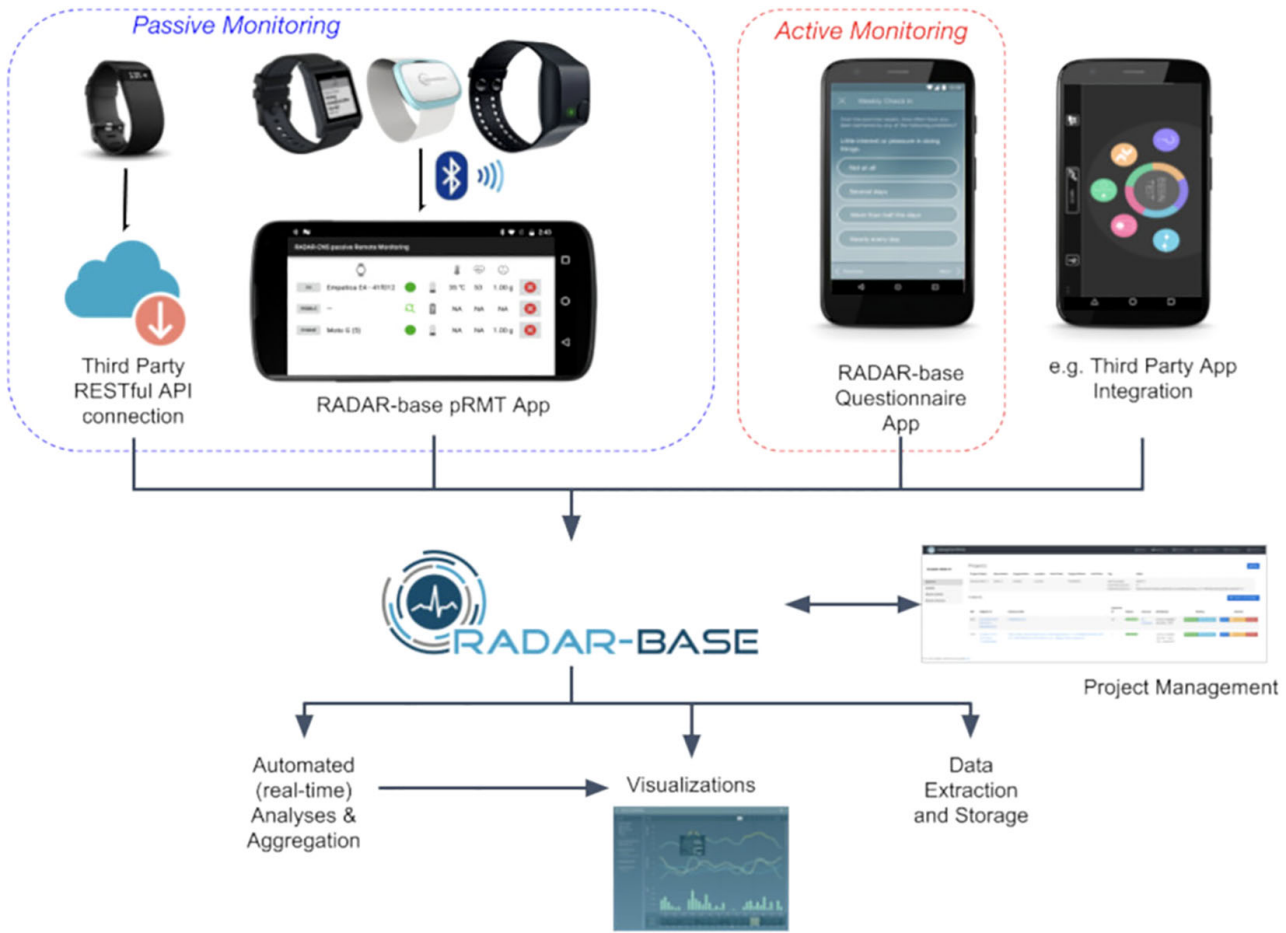
Overview of the “Radar-Base” program, a home monitoring program for patients showing key digital components such as wireless spirometry, activity tracking, PROs, and communication tools. This model supports remote monitoring approaches by monitoring patients remotely with great self-management (54).

Moreover, the primary limitation of home-based spirometry is the patient’s adherence to the instructions, which can vary over time for the same patient. For example, adherence to spirometry, which was used twice weekly, was moderate (53%) in the rural cohort population study conducted by Boente et al., whereas several other studies reported 70%-90% adherence rates (37, 52, 53). In comparison to hospital visits, patients certainly do not see it as an alternative to hospital visits, but as an additional supporting approach, as the study by Velauthapillai et al.: “*No, I would like to receive guidance in the hospital and also appreciate the hospital measurements more*” (50). Additionally, as seen in **Figure 9**, several current programs are specialized for a specific disease, such as the sarcoidosis program “SarcOnline” (55).

Moreover, several significant guidelines have recently been published that have a high potential to guide the clinical management of CTD-ILD. In 2023, the ACR/CHEST guidelines on screening and monitoring issued 15 recommendations based on 24 PICO questions. While the other guidelines on treatment included 35 recommendations developed from 216 PICO questions. In 2023, the ATS guidelines for SSc-ILD covered 8 key questions that resulted in 6 treatment and 2 research recommendations. More broadly, the joint ERS/EULAR guidelines addressed 50 recommendations that integrates both structured PICO-based evidence and 26 narrative questions, spanning screening, diagnosis, treatment, and follow-up. Together, these guidelines provide a much-needed evidence-based framework for the clinical practice. However, a clear gap still remains. In detail, despite the comprehensive scope, digital health and remote monitoring are still not meaningfully addressed. In fact, this lack highlights the opportunity to bring emerging digital tools, to complement and extend guideline-based care in CTD-ILD.

To address and overcome those challenges, An important recent study by Khor et al. addresses an essential structured approach, the PANACEA framework, as a new guidance evaluating the RPM systems if meeting the conditions for clinical and research use and its suitability for implementation in chronic lung disease, including RA-ILDs (56).

**Figure 10:**
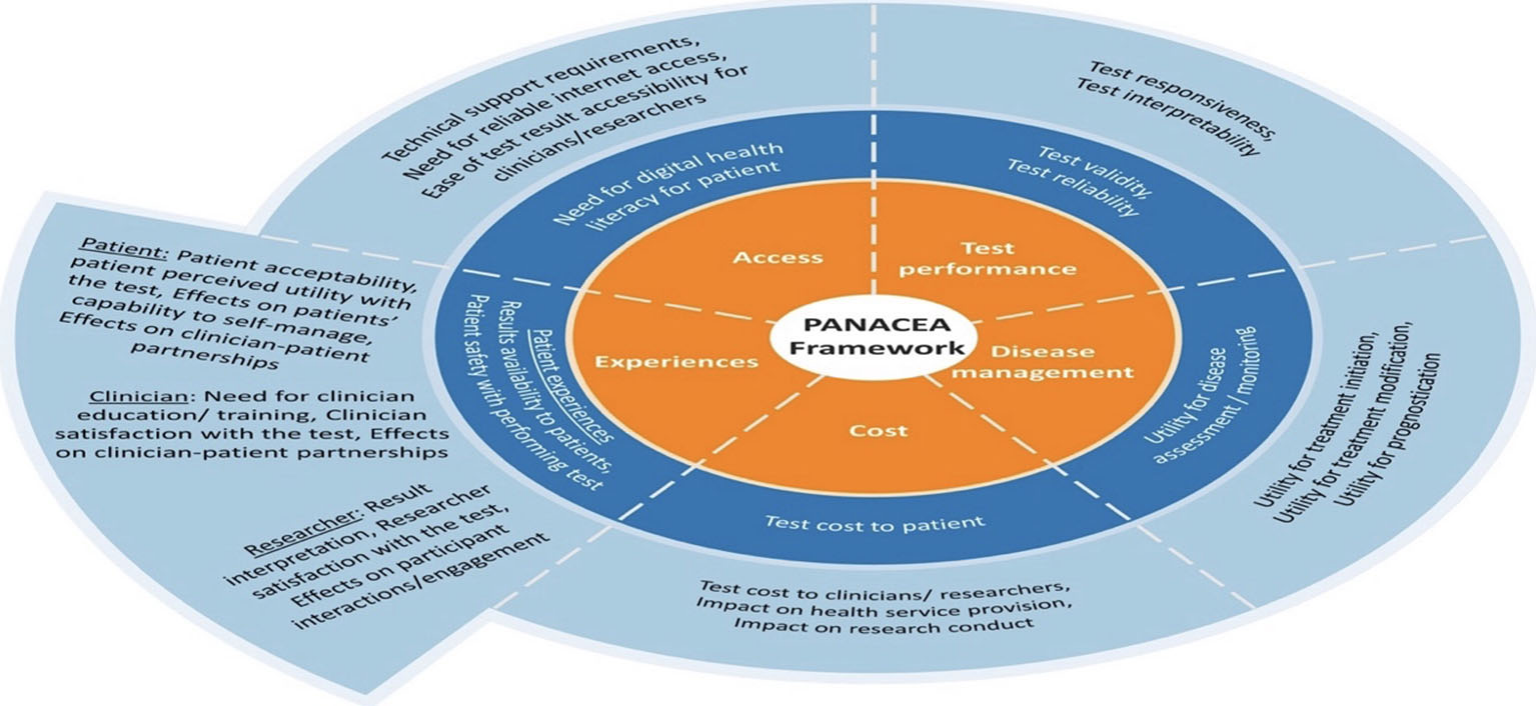
The PANACEA framework, the multidimensional framework for evaluating home-based monitoring tests in chronic lung disease. This conceptual framework outlines five interrelated domains: test performance, disease management, costs, experiences, and access that influences the evaluation and implementation of digital health interventions. At the center, the framework integrates the domains to guide a comprehensive assessment approach. the outer layers specify the factors related to each domain. This framework emphasizes the importance of stakeholder engagement and multidimensional evaluation to optimize the development and impact of RPMsystems.^[98]^.

The PANACEA framework, with its numerous domains, focuses on assessing several essential items, as seen in **Figure 10**. Those essential domains include test performance, disease management, cost, patient experience, clinician experience, researcher experience, and access. This framework has also been successful in addressing the knowledge gaps and providing recommendations for enhanced future research to improve the quality of home-based monitoring for both patients and clinicians, which promises strong, valid recommendations for future home-based programs to achieve high-potential outcomes in clinical practices (56).

## Digital health: from today to the future

**Figure 11:**
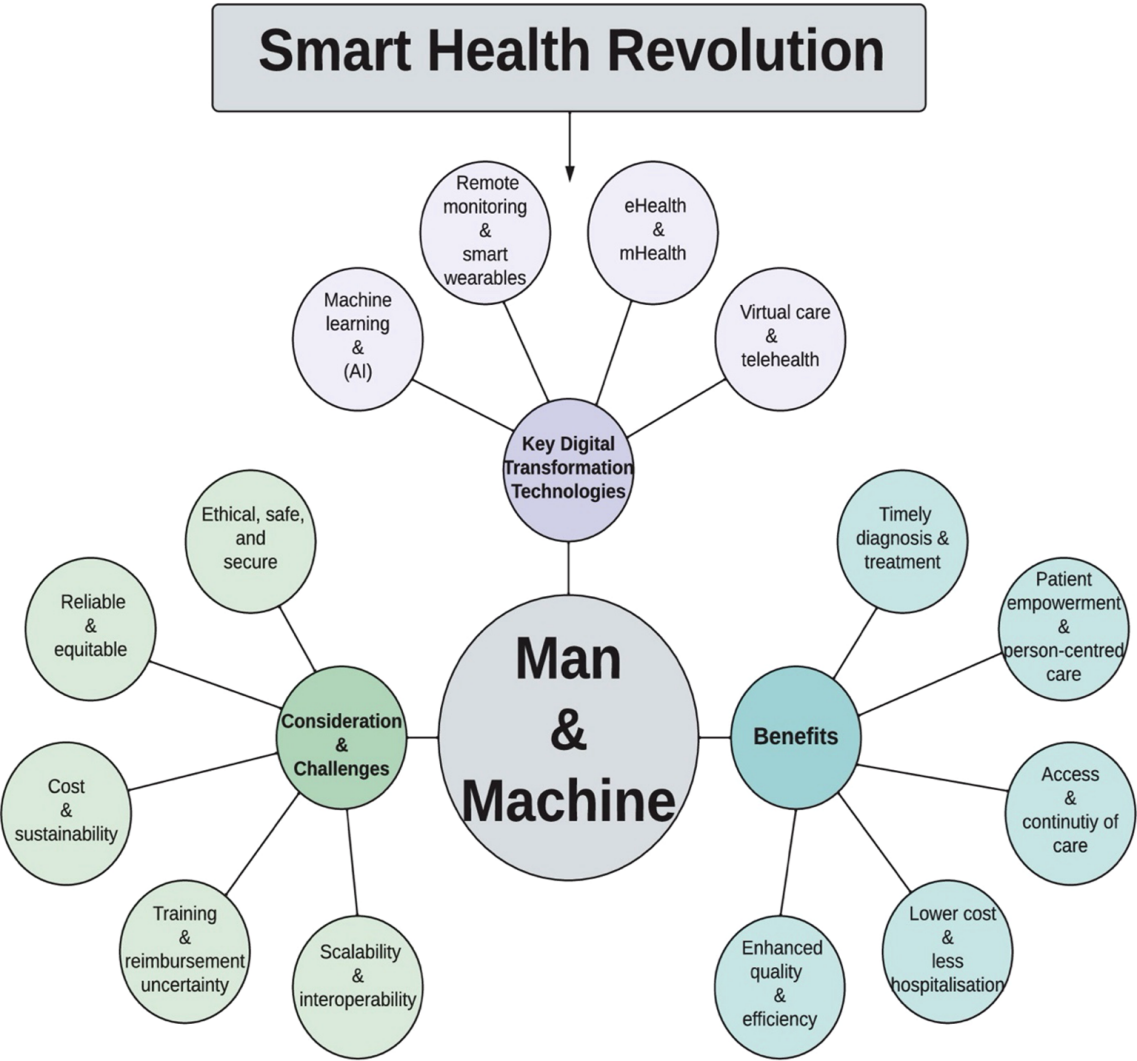
The Smart Health Revolution: Overview of Human–Machine Partnership in Digital Health. This conceptual diagram visualizes the evolving synergy between “man”, which refers to the patient/clinician, and “machine”, which refers to digital health technologies. The core represents the key digital transformation technologies (remote monitoring and wearables, eHealth/mHealth, telehealth/virtual care, and machine learning/AI). Besides illustrating the most significant benefits, such as patient empowerment, enhanced efficiency, timely diagnosis, reduced hospitalizations, and access to and continuity of care. In addition to considerations and challenges, including cost and sustainability, equitable access, interoperability/scalability, training and reimbursement, ethical and safe implementation (57).

The illustration in **Figure 11** retains the spirit of the promising collaboration between the human element in clinical practice, whether the clinicians or the patients themselves, and the current intelligent machines, resembling the digital health and its great role currently and for future promises. Significantly, it conceptualizes the central role that the provided care for respiratory disease patients is best not as a purely humanized arm, but as a technological alliance between patient, clinician, and machine (57).

While recentring it in the context of remote monitoring in autoimmune‐associated ILD, the framework’s benefits side, those technologies promise faster diagnoses, smoother continuity of care, empowered patients in disease management, lower disease and healthcare burdens, and better care efficiency. In contrast, these benefits demand real-world considerations and challenges, including the cost of scaling, ensuring fairness in access, integrating with fragmented health systems, defining reimbursement mechanisms, and designing ethically robust and safe tools.

Most importantly, what gives this model its significance is its fostering of imagining a future of automation and responsive collaboration, where digital health extends rather than replaces empathy, and enriches rather than replaces the clinician–patient relationship. Thus, it aligns with the core argument of this review: that the promise of digital health in RA-ILDs lies in its capability to improve the clinician–patient relationship, providing continuous personalized care for patients with RA-ILDs.

## Conclusion

Autoimmune related ILD (AR-ILD) remains a major cause of morbidity and mortality, demanding early recognition, multidisciplinary care, and tailored therapeutic strategies. Because trajectories are unpredictable and current strategies only slow progression, integrating digital monitoring enables earlier detection and personalized care. Therefore, integrating digital medicine and remote monitoring strategies into routine practice offers a promising path for effective patient follow-up to detect progression, personalize treatment, and address the broader physical and emotional burden of disease. Thus, transforming episodic, clinic-based care into continuous, patient-centered management.

Evidence is increasingly supporting home spirometry, wearable devices, and telerehabilitation as acceptable, feasible, and impactful in improving both physiological monitoring and emotional well-being. These emerging tools, though not yet standard, are reshaping patient engagement and care delivery. However, several challenges in usability, adherence, cost, and equity highlight the need for validation and implementation. Future research should prioritize the integration of these tools into standard care, explore their impact on long-term outcomes, and ensure accessibility across diverse clinical practices, as their continued refinement and validation could redefine long-term management in this high-risk population. By embedding digital monitoring more in clinical practices, we can move toward a more personalized, proactive, and equitable model of RA-ILD care that redefines patient engagement and reshapes disease trajectories.
